# Combined intravenous ribavirin and recombinant human interferon α1b aerosol inhalation for adenovirus pneumonia with plastic bronchitis in children: a case report and review of literature

**DOI:** 10.3389/fped.2024.1295133

**Published:** 2024-02-06

**Authors:** Liangkang Lin, Maoting Tang, Deyuan Li, Haotian Fei, Haiyang Zhang

**Affiliations:** ^1^ Department of Pediatrics, The Eighth Affiliated Hospital, Sun Yat-Sen University, Shenzhen, China; ^2^ Department of Pediatrics, West China Second University Hospital, Sichuan University, Chengdu, China; ^3^ Key Laboratory of Birth Defects and Related Diseases of Women and Children of Sichuan University, Ministry of Education, Chengdu, China; ^4^ Department of Pharmacy, West China Second University Hospital, Sichuan University, Chengdu, China

**Keywords:** human adenovirus, pneumonia, plastic bronchitis, ribavirin, children, case report

## Abstract

**Background:**

Human adenovirus (HAdV) infections in children can lead to profound pulmonary injury and are frequently associated with severe complications, particularly in cases concomitant with plastic bronchitis. Managing this condition presents significant challenges and carries an exceptionally high fatality rate. Regrettably, there are currently no specific antiviral agents that have demonstrated efficacy in treating severe adenovirus pneumonia in children.

**Case presentation:**

We report a 10-month-old infant suffering from severe adenovirus pneumonia combined with plastic bronchitis (PB). He received intravenous ribavirin combined with recombinant human interferon α1b (INFα1b) aerosol inhalation and his condition eventually improved. No side effects occurred during the treatment, and the long-term prognosis was favorable.

**Conclusion:**

In this case, the combination therapy of intravenous ribavirin and INFα1b seems to have contributed to the resolution of illness and may be considered for similar cases until stronger evidence is generated.

## Introduction

Human adenovirus (HAdV) is a prevalent community-acquired virus associated with lower respiratory tract infections, manifesting as various clinical presentations including otitis media, conjunctivitis, and myocarditis. Throughout various countries, the implementation of public health and social measures (PHSMs) has resulted in varying trends in the prevalence of HAdV (1). Despite these measures, the incidence of HAdV has persistently remained elevated among pediatric populations. In the milieu of the COVID-19 pandemic, global data indicate that the prevalence of HAdV in children spans from 6.9% to 15.8% (2). The most critical manifestation of HAdV infections in children typically involves the lungs (3). Adenovirus pneumonia can rapidly progress to refractory respiratory failure, posing a life-threatening condition. Furthermore, severe adenovirus pneumonia can be complicated by additional conditions such as plastic bronchitis (PB), bronchiectasis, and occlusive bronchiectasis, which further exacerbate lung damage and prolong illness duration. The reported mortality rate for children with adenovirus pneumonia ranges from 0.38% to 20% in severe cases (4).

Currently, the treatment principle for adenovirus pneumonia in children is symptomatic treatment to improve symptoms and supportive treatment to protect organs, including respiratory support, airway clearance techniques, glucocorticosteroids, intravenous immunoglobulin, and extracorporeal membrane oxygenation (ECMO) (5). At present, there is a lack of specific anti-adenovirus drugs for the etiological treatment of adenovirus pneumonia. Limited research has been conducted worldwide on antiviral drugs for the treatment of HAdV infections. Adenovirus pneumonia combined with PB in children may greatly worsen the prognosis, and the traditional symptomatic supportive treatment will become less effective. There is an ongoing need to develop effective etiological treatment strategies for severe adenovirus pneumonia in children, including reassessing the effectiveness and safety of classic antiviral drugs such as ribavirin, to improve the cure rate. In this article, we reporton an infant suffering from severe adenovirus pneumonia combined with PB. To achieve successful recovery, a clustered treatment strategy was implemented involving intravenous ribavirin combined with nebulized recombinant human interferon α1b (INFα1b). This is the first report describing the utilization of intravenous ribavirin combined with nebulized recombinant human INFα1b for the treatment of severe adenovirus pneumonia combined with PB in children. This case report can provide valuable clinical experience for the treatment of severe adenovirus pneumonia in children and highlight the potential benefits of intravenous ribavirin.

## Case presentation

**Figure 1:**
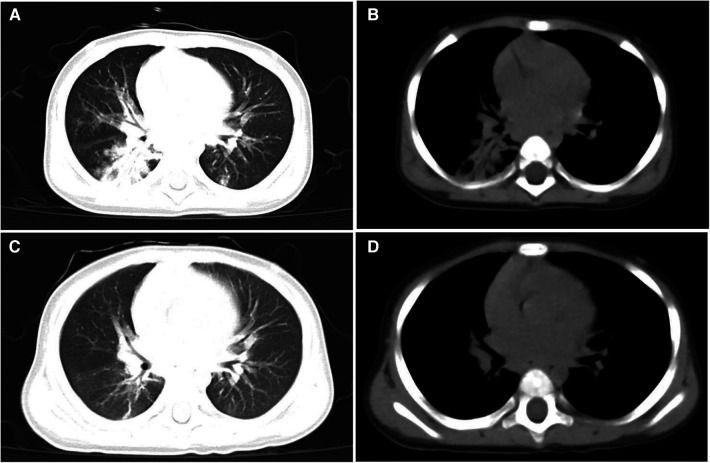
Chest CT scanning of the patient. CT lung window on admission showed double pneumonia with partial consolidation of the lower lobe of the right lung (**A**) CT mediastinal window on admission showed slight enlargement of bilateral hilar lymph nodes (**B**) CT lung window on the 30th day of admission showed a small amount of lung inflammation and a few pulmonary interstitial changes (**C**) CT mediastinal window on the 30th day of admission did not show pulmonary lymph node enlargement anymore (**D**).

A male infant, aged 10 months, was admitted to the Pediatric Intensive Care Unit (PICU) of West China Second Hospital in August 2022. He presented with chief complaints of recurrent fever, cough, and shortness of breath persisting for 6 days, and cyanosis observed for 1 day. The fever was characterized as moderate to high, and the cough, noted as severe and productive, was associated with aspiration during feeding. The child was born at full term and has been breastfed ever since. He had no history of congenital heart disease, COVID-19, influenza virus, respiratory syncytial virus, or HAdV infection. On admission, physical examination revealed a temperature of 38.3°C, heart rate of 184 beats/min, respiratory rate of 57 times/min, blood pressure of 105/73 mmHg, blood oxygen saturation of 90% (5l /min oxygen flow via a mask), cyanosis of the face and lips, nasal flaring, and a positive inspiratory triple concavity sign. Upon auscultation, numerous coarse, moist rales and expiratory wheezing sounds were audible in both lungs. The fontanel was neither depressed nor bulging. Physical examination of the cardiovascular, digestive, and nervous systems revealed no abnormalities. Arterial blood gas (ABG) analysis revealed a pH of 7.408, partial pressure of oxygen (paO_2_) at 36.2 mmHg, partial pressure of carbon dioxide (paCO_2_) at 36.2 mmHg, oxygen saturation (SaO_2_) at 59.4%, bicarbonate (HCO_3_^−^) at 22.8 mmol/L, and lactate levels measured at 3 mmol/L. Routine blood examination showed white blood cells (WBC) were 13.0 × 10^9^/L, neutrophil percentage was 80.8%, blood platelet (PLT) was 434 × 10^9^/L, hemoglobin (HGB) was 127 g/L. C-reactive protein (CRP) was 28.2 mg/L, and procalcitonin was 2.2 ng/ml. Myocardial injury markers, liver and kidney function, coagulation parameters, echocardiography, and electrocardiogram were all within normal limits. Chest computed tomography (CT) showed double pneumonia with partial consolidation of the lower lobe of the right lung (lung window, Figure 1A) and slight enlargement of bilateral hilar lymph nodes (mediastinal window, Figure 1B). The initial diagnosis for the patient indicated severe pneumonia accompanied by respiratory failure.

**Figure 2:**
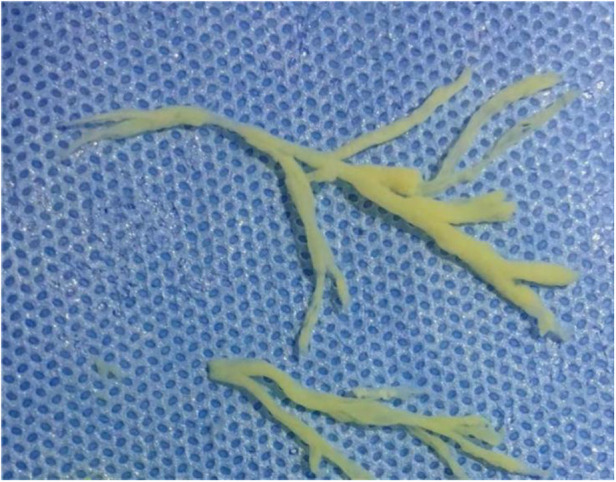
Numerous of white plasmapheresis plugs was removed by bronchoscopic clamp.

**Figure 3:**
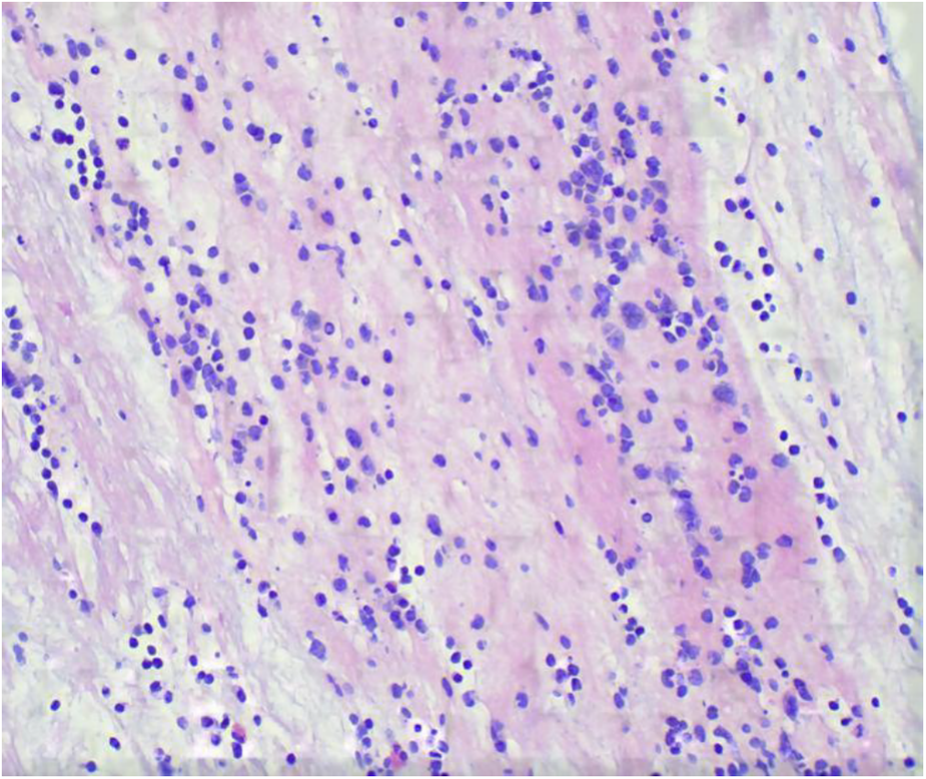
Pathology of bronchoplastic sputum suppositories showed exudate fibrin and mucus-like material with more inflammatory cells and histiocytic infiltration (HE stains, ×400).

Upon admission, he underwent bi-level positive airway pressure (BiPAP) ventilation for assisted breathing. Meanwhile, he was treated with meropenem (60 mg/kg/day, q8h, for 14 days) for empiricalanti-infective therapy, methylprednisolone (3 mg/kg/day, q8h, for 3 days) to fight inflammation, albuterol combined with budesonide aerosol inhalation, intravenous gamma globulin (1 g/kg) to boost immunity, and ambroxol hydrochloride for expectoration. These interventions can temporarily relieve shortness of breath. On the 2nd day of admission, his condition was worsened by a persistent high fever and increased viscous airway secretions. We added vancomycin (40 mg/kg/day, q6h, for 14 days) to empirically treat Methicillin-resistant *Staphylococcus aureus* (MRSA) infection. On the 3rd day of admission, the blood culture and the sputum culture were negative. The polymerase chain reaction (PCR) of the throat swab for multiple respiratory pathogens showed HAdV was positive and *bordetella pertussis*, mycoplasma pneumoniae, influenza virus, boca virus, respiratory syncytial virus, and COVID-19 were negative. The serum G [(1–3)-β-d-glucan] test and galactomannan (GM) test were negative. The metagenomic next-generation sequencing (mNGS) of blood revealed the presence of HAdV with specific sequences identified as 74,376 reads, showing high confidence. On the 4th day of admission, the SaO_2_ dropped again (85%–90%), and a reexamination of chest x-ray revealed increased lung consolidation. The ventilation mode was promptly switched to invasive ventilator-assisted ventilation, and a large amount of yellowish-white viscous sputum was sucked out of the tracheal tube. Fiberoptic bronchoscopy and bronchoalveolar lavage were performed. The white jelly dendritic obstruction in the left upper and lower bronchial lumens was observed by fiberoptic bronchoscope. No bleeding or new organisms were observed. Immediately, a bronchoscopic clamp was performed to remove numerous white plasmapheresis plugs (Figure 2). The pathology of bronchoplastic sputum suppositories showed exudate fibrin and mucus-like material with more inflammatory cells and histiocytic infiltration (Figure 3). Laboratory results of bronchoalveolar lavage fluid (BALF) were as follows: nucleated cell count of 160 × 10^6^/L, with neutrophilic lobulated nucleated granulocytes accounting for 29% and lymphocytes accounting for 16%. No abnormalities were observed in acid-fast staining, silver hexamine and PAS staining, or alveolar lavage fluid culture. On the 6th day of hospitalization, the mNGS of the BALF revealed the presence of HAdV with specific sequences identified as 223,049 reads and high confidence. On the 8th day of admission, due to the patient's lung inflammation still progressing, we organized a multi-disciplinary treatment (MDT) involving the department of Pediatric Infectious Diseases and the department of Clinical Pharmacology. Despite the administration of intensive treatments including glucocorticoids, intravenous gamma globulin, and fiberoptic bronchoscopy, the patient's pneumonia condition deteriorated. Concurrently, mNGS of the BALF and blood samples indicated high load HAdV infection in this case. To prevent adenovirus replication and further damage to the lungs, we administered intravenous ribavirin (7.5 mg/kg, q12h, for 7 days, each infusion time >20 min) combined with INFα1b atomization (4 ug/kg/day, q12h, for 7 days) for antiviral therapy. On the 10th day of admission, the temperature remained below 37.5°C. We gradually lowered the ventilator parameters, and the patient's oxygen saturation could still be maintained above 95%. On the 12th day of admission, the demand for oxygen further decreased, and we lowered the ventilator parameters again. On the 14th day of admission, chest x-ray showed a significant reduction in lung consolidation. We replaced meropenem and vancomycin with intravenous ceftriaxone for sequential anti-infection (100 mg/kg/day, q12h, for 14 days). On the 16th day of hospitalization, the PCR of HAdV turned negative and the ABG analysis were completely normal. Mechanical ventilation was withdrawn and replaced with nasal high-flow oxygen therapy (8 L/min oxygen flow). Meanwhile, ribavirin and INFα1b were discontinued. On the 28th day of hospitalization, a repeat fiberoptic bronchoscopy revealed a smooth endothelial lining of the bronchial tubes with a small amount of thin white secretion, and no bronchial sputum plugs or plasmapheresis were observed. The ventilation mode was changed to nasal catheter oxygen inhalation (0.5 L/min oxygen flow) and switched to oral cefaclor (30 mg/kg/day, for 7 days) instead of intravenous ceftriaxone. On the 30th day, his oxygen saturation remained above 95% without oxygen intake, and there were no signs of fever, shortness of breath, or mental deterioration. The physical examination found that the positive signs in the patient's lungs had disappeared. The reexamination of chest CT revealed only a small amount of lung inflammation and a few pulmonary interstitial changes (lung window, Figure 1C), while it did not show pulmonary lymph node enlargement (mediastinal window, Figure 1D). Consequently, the infant was discharged from the hospital successfully and continued to take oral cefaclor for an additional 3 days after discharge. During the entire hospitalization period, serial weekly assessments of liver function, renal function, myocardial injury markers, and hemoglobin levels consistently yielded normal results. Additionally, no instances of rash or convulsion were observed.

**Figure 4:**
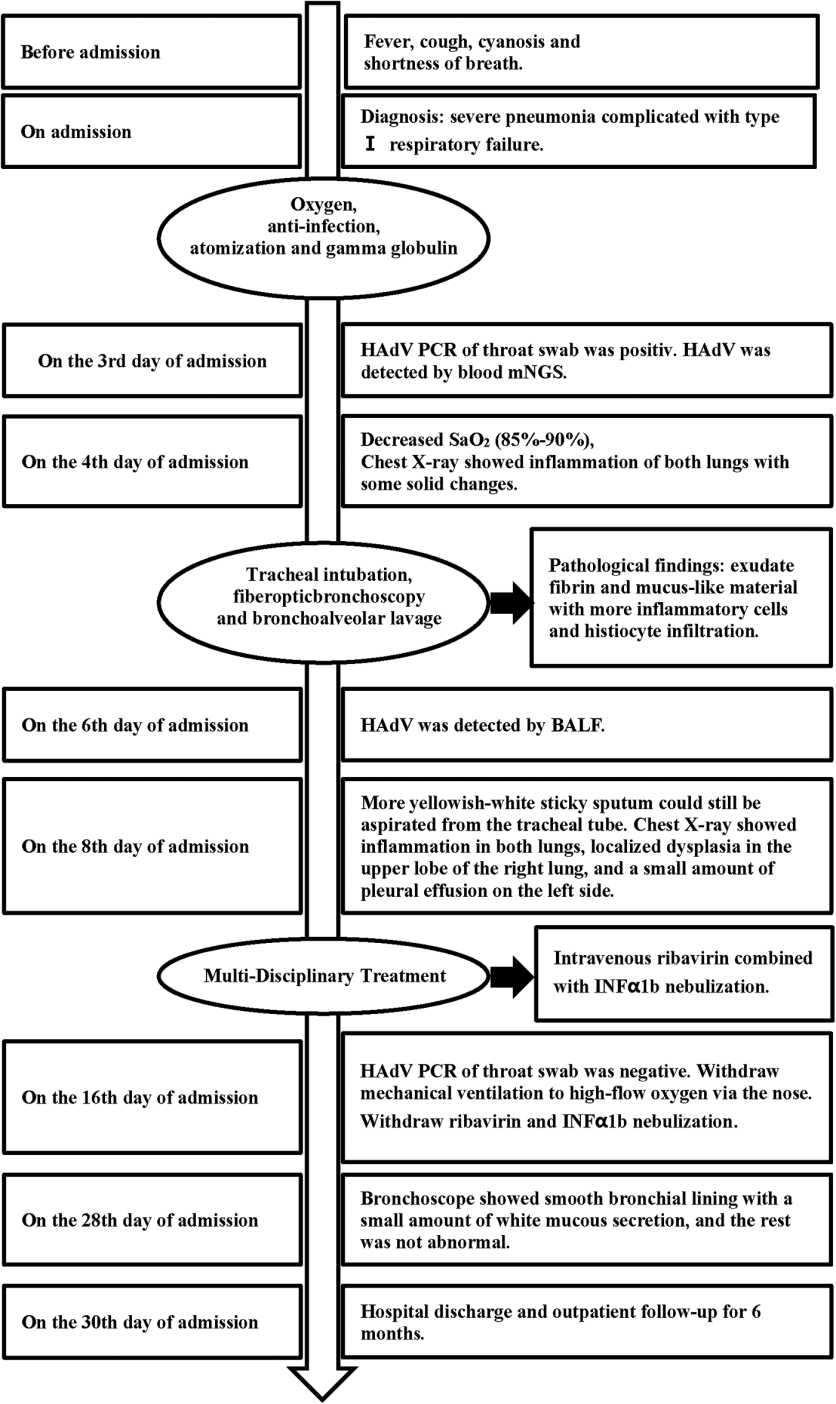
The timeline of disease progression and treatment.

During the 6-month follow-up in the outpatient clinic, there were no instances of HAdV infection recurrence. Symptoms of recurrent coughing and milk choking did not appear. ABG analysis, blood routine, liver function, renal function, markers of myocardial injury, and hemoglobin indexes were all within normal ranges. The pulmonary function test and hearing screening showed normal results. Two repeat chest CT scans during follow-up revealed no imaging signs of solid lung lesions, interstitial lung changes, pulmonary atelectasis, or bronchiectasis. The timeline of treatment and progression is summarized in Figure 4.

## System review

A thorough review of the literature from 1970 to 2023 was undertaken, utilizing databases such as PubMed, Web of Science, Embase, and Medline. This search employed keywords including “adenovirus”, “intravenous infusion”, “ribavirin”, “plastic bronchitis”, and “children”. From the selected articles, pertinent information was extracted, encompassing the first author's name, publication year, country of study, patient age range, underlying conditions, identified risk factors, treatment modalities, duration of treatment, observed drug side effects, and treatment outcomes. In Table 1, we have compiled data from six published articles detailing the administration of intravenous ribavirin for treating HAdV infection in pediatric patients (6–11). Additionally, Table 2 presents a synthesis of six published articles focusing on pediatric cases of adenovirus pneumonia with concurrent PB (12–17).

**Table 1:** Published articles on pediatric cases treated with intravenous ribavirin for HAdV infection.

Author	Country/year	Male/female	Age	Underlying disease	Risk factor	Duration of treatment	Ribavirin loading dosage	Ribavirin maintenance dosage	Side effect	Recovery rate
Gavin et al. (4)	The United States, 2002	2/3	8 days–18 years	Cystitis, nephritis, disseminated, pneumonia	OHT, AML/BMT, Neonate, DiGeorge	Undisclosed	25–33 mg/kg/day	8–15 mg/kg/day	None	40%
Murphy et al. (5)	The United States, 1993	1/0	8 years	Cystitis, nephritis	AML/BMT	8 days	35 mg/kg/day	25 mg/kg/day	None	100%
McCarthy et al. (6)	The United States, 1995	Undisclosed	14 months	Disseminated	ALL	Undisclosed	30 mg/kg/day	15 mg/kg/day	None	100%
Pichler et al. (7)	Germany, 2000	Undisclosed	6 weeks, 16 years	Bronchiolitis	None	10 days	30 mg/kg/day	15 mg/kg/day	None	50%
Bertrand et al. (8)	The United States, 2000	0/1	8 years	Pneumonia	Multivisceral transplantation	>10 days	35 mg/kg/day	8–16 mg/kg/day	Hyperammonemia	0%
Kapelushnik et al. (9)	Israel, 1995	1/0	3 years	Gastroenteritis	Wiscott-Aldrich, BMT	10 days	30 mg/kg/day	20 mg/kg/day	None	100%

OHT, orthotopic heart transplant; AML, acute myeloid leukemia; BMT, bone marrow transplant; ALL, acute lymphoblastic leukemia.

**Table 2:** Published articles on pediatric cases of adenovirus pneumonia with concurrent PB.

Author	Country/year	Male/female	Age	Duration of treatment	Treatment	Bronchoscope	Antiviral drug	Recovery rate
Zhang et al. (12)	China, 2022	2/1	45 months–63 months	25–45 days	Antibiotic, hemodialysis, corticosteroid, mechanical ventilation, immunosuppressant, ECMO	Done	None	33.3%
Huang et al. (13)	China, 2022	Undisclosed	7 months–10 years	12 (7–25) days	Antibiotic, corticosteroid, immunoglobulin	Done	None	Undisclosed
Zeng et al. (14)	China, 2021	4/6	10 months–15 years	11.38 ± 3.335 days	Antibiotic, corticosteroid, immunoglobulin	Done	None	90%
Yuan et al. (15)	China, 2020	1/1	2 years, 3years	Undisclosed	Antibiotic, corticosteroid, immunoglobulin	Done	None	100%
Zhang et al. (16)	China, 2020	0/1	4 years	39 days	Antibiotic, corticosteroid, immunoglobulin, ECMO	Done	None	100%
Lu et al. (17)	China, 2018	1/0	3 years	Undisclosed	Undisclosed	Done	None	100%

PB, plastic bronchitis; ECMO, extracorporeal membrane oxygenation.

## Discussion

HAdV belongs to the adenoviridae and is a medium-sized (70–100 nm) enveloped virus. It possesses icosahedral capsids that enclose a double-stranded linear DNA genome ranging from 34 to 36 kbp in length (3). This virus is primarily transmitted through respiratory droplets and exhibit rapid and efficient spread among children. Adenovirus pneumonia in children manifests with a wide range of clinical presentations, spanning from mild respiratory infections to severe pneumonia. The disease course is characterized by rapid progression, and it can give rise to severe complications such as PB. PB often leads to tracheal obstruction and lung function damage, which eventually cause respiratory failure and death. The mortality rate of infection-induced inflammatory plastic bronchitis has been reported to be between 6% and 50% (18).

Human airway epithelial cells serve as suitable hosts for HAdV proliferation and replication. Severe HAdV infection can cause airway epithelial shedding and the infiltration of inflammatory cells and mucus in the airway, leading to plastic bronchitis. It has been reported that the pathogenesis is adenovirus activation of the immune response mediated by macrophages, natural killer cells, monocytes, and type 1 IFNα1b in respiratory tract columnar epithelial cells (19). This activation induces the release of complement C3a and IL-13, subsequently resulting in the upregulation of genes such as mucin 5B and MUC5AC (20). These gene upregulations promote excessive mucus secretion and the formation of PB. Once adenovirus infection is not effectively controlled in the early stages, it can continuously induce the production of secretion-promoting factors and oxygen free radicals, stimulating the proliferation of secretory cells and leading to excessive mucus accumulation in the airway. This, in turn, causes airway obstruction, airflow limitation, reduced cilia clearance, and alterations in the physicochemical properties of mucus. The accumulation of airway mucus creates a conducive environment for the growth of viruses and bacteria (21). Based on the above pathophysiological mechanism, it is crucial to combine immunomodulatory therapy (glucocorticoids, gamma globulin) and airway clearance techniques for severe adenovirus pneumonia. The former helps reduce the risk of bronchial plastination and the recurrence of cytokine storms, while the latter aids in alleviating airway obstruction and restoring ventilation function. However, due to the extensive replication of adenovirus in the body, the effectiveness of the aforementioned intensive treatment regimen is limited to the symptomatic management of airway complications in severe adenovirus pneumonia. In severe cases, curing the disease and avoiding plastic bronchitis recurrence can be achieved by directing etiological treatment toward the infectious pathogen.

Currently, the treatment options for HAdV infection in children are extremely limited. There is no specific antiviral drug that effectively targets adenovirus. Although some drugs, such as ribavirin and cidofovir, possess broad-spectrum antiviral activity, they are not specifically designed for HAdV infection. Ribavirin, a synthetic nucleoside antiviral, inhibits the activity of hypoxanthine nucleotide dehydrogenase, resulting in a potent inhibitory effect on various DNA and RNA viruses (22, 23). Additionally, it can reduce the excessive immune response and organ dysfunction caused by viral infection by reducing cytokine production and the inflammatory response (24). Some studies have indicated the use of ribavirin in the treatment of HAdV infection in immunocompromised children, such as patients who have received stem cell transplantation, solid organ transplantation, or chemotherapy for malignant tumors (25). These children are more susceptible to severe HAdV infection due to their weakened immune systems, making ribavirin a viable treatment option.

For immunologically normal children, ribavirin is often employed as adjunctive therapy for community-acquired viral infections through aerosol inhalation and oral administration, with less frequent intravenous use. The application of intravenous ribavirin is constrained by multiple factors, predominantly due to apprehensions regarding potential toxic side effects stemming from an unregulated intravenous route. Unregulated intravenous ribavirin may cause anemia, hypocalcemia, abnormal liver function, hemolysis, hypomagnesemia, and lymphopenia. These drug toxicities accounted for 36.4%, 24.1%, 15.4%, 12.9%, 11.3%, and 10.8% of the total reported toxic side effects of intravenous ribavirin, respectively (26). The occurrence of these side effects is dose-dependent and related to an excessive course of treatment. To reduce the risk of organ function impairment due to prolonged drug accumulation, most cases in the literature implemented initial loading dose and post-loading maintenance doses of ribavirin for a limited treatment duration of 14 days. This scheme can not only ensure the efficacy of the drug, but also avoid the organ function damage caused by the accumulation of the drug in the body for a long time. In China, the recommended intravenous infusion dose of ribavirin for children is 10–15 mg/kg/day, administered in two divided doses with each intravenous drip lasting more than 20 min. The course of treatment is recommended for 3–7 days, but the initial ribavirin loading dose is not required. Among the case reports reviewed, only one case had the drug side effect of hyperammonemia due to intravenous ribavirin. The remaining reported cases of intravenous ribavirin have achieved favorable cure rates and no toxic side effects. These findings further support the efficacy and safety of the drug in the treatment of HAdV infection in children. We prioritize the safety of intravenous ribavirin combined with INFα1b aerosol therapy. Rigorous monitoring for adverse events constitutes a crucial aspect of our treatment protocol. Specifically, we meticulously recorded the patient's vital signs. Concurrently, we maintained continuous vigilance over the patient's blood routine, liver function, electrolyte, and other biochemical indicators to detect potential drug-related adverse reactions. During ribavirin use, these indicators were tested twice a week. It is noteworthy that, in our cases, no adverse events related to ribavirin and INFα1b treatment were observed. Nevertheless, we stress that the absence of observed adverse events does not imply the absolute safety of this treatment regimen.

Intravenous ribavirin allows for rapid delivery of the drug to the target tissues and organs, leading to faster antiviral effects compared to oral administration (27, 28). Ribavirin remains an approved option for viral infections worldwide, including those without specific antiviral drugs, due to its broad antiviral activity (29). However, there is limited clinical experience with ribavirin intravenous for the treatment of HAdv infection in children. We had reviewed only six relevant case reports. The patient profiles described in the literature review exemplify the subset of immunocompromised pediatric populations most vulnerable to severe adenovirus infections, particularly solid-organ and bone marrow transplant recipients, neonates, and children with immunodeficiencies. Pichler et al. (7) reported two cases of severe bronchitis in healthy children. Both patients were treated with intravenous ribavirin for 10 days. Ultimately, the patient who underwent bronchoalveolar lavage was cured, while the other died. INFα1b is an immunomodulator with antiviral and immunomodulatory effects (30). Its antiviral activity is primarily achieved by inducing the expression of the INFα1binterferon-stimulated gene ISG and antiviral proteins. It also restricts early adenoviral replication through the interferon-induced protein tetratripeptide repeat sequence 3 protein IFIT3, also known as ISG 60 (31). Combining ribavirin and INFα1b can enhance therapeutic efficacy by simultaneously targeting different stages of HAdV proliferation. While the combination of ribavirin and INFα1b has been successfully used in the treatment of hepatitis C virus (HCV), their combined drug experience in HAdV treatment has not been extensively reported. However, an *in vitro* experimental study has demonstrated that the combination of ribavirin and INFα1b promoted higher cell survival rates compared to ribavirin alone. The inhibitory effect of the combination was achieved at lower concentrations than the individual drugs alone at higher concentrations, suggesting synergistic effects (32). Additionally, the combination of ribavirin and INFα1b resulted in the upregulation of signal transducer and activator of transcription (STAT) 1 and STAT2, IFN regulatory factors (IRF) 9, and ISG 15 expression, as well as increased phosphorylation levels of STAT1 and STAT2, indicating the potential involvement of ribavirin in the janus kinase/signal transducer and activator of transcription (JAK/STAT) signaling pathway (33). Combining intravenous ribavirin with INFα1baerosol inhalation enhanced the expression level, stability, and durability of INFα1b as well as prolonging its antiviral activity (24). In contrast to the literature review, this case involved a rapidly progressing immunocompetent child with the challenging complication of PB. We employed treatment strategies including intravenous ribavirin in combination with INFα1b and timely bronchoscopic airway contouring techniques, resulting in a successful cure with no adverse drug reactions. Although similar pediatric cases of intravenous ribavirin are currently rare in clinical practice, it can highlight the potential effectiveness and feasibility of intravenous ribavirin as a therapeutic option for severe adenovirus pneumonia in children.

In the management of severe adenovirus pneumonia complicated by PB, the current conventional treatment involves providing adequate respiratory support and performing early fiberoptic bronchoscopy to remove the plastic phlegm plug. Earlier fiberoptic bronchoscopic alveolar lavage and bronchial plastic extraction can effectively treat airway obstruction. The articles presented in Table 2 document the application of bronchoscopy in treating this clinical condition, with outcomes indicating favorable results (13–17). However, treatment with fiberoptic bronchoscopy alone leads to the risk of recurrent exacerbations of PB or the development of occlusive bronchiectasis (34). For severe inflammatory PB, the persistence of the virus may result in ongoing inflammation and lesions in the airways, which predisposes to recurrent exacerbations of PB during the treatment course. In Table 2, the fatal case reported by Zhang D et al. (12) is detailed, illustrating that despite the immediate intervention with fiberoptic bronchoscopy for the removal of the plastic sputum plug, the patient's condition progressively worsened. This raises a critical question regarding the potential role of ongoing adenovirus infection in the deterioration of the child's health. Based on the successful experience of this case, we believe that intravenous ribavirin for antiviral therapy, along with the control and reduction of airway inflammation, can reduce the formation of plastic phlegm suppositories and effectively prevent recurrence when combined with timely bronchoscopic therapy.

Despite the successful experience reported in this case of using intravenous ribavirin for the treatment of severe adenovirus pneumonia in children, the limitations of the case report need to be considered. First of all, we were unable to prove from a single case report that the combination therapy used in this case is the reason why this child resolved the HAdV infection. Each patient's condition may vary, including age, severity of illness, and immune status, which may affect treatment outcomes and the occurrence of side effects. When using intravenous ribavirin to treat HAdV infection in children, strict adherence to the prescribed dosage should be emphasized, while the patient's condition and drug response should be closely monitored. The potential clinical benefits and risks of ribavirin need to be dynamically evaluated during medication. To sum up, in order to demonstrate a positive effect of ribavirin and interferon, a more robust investigation should be carried out, and this case report only generates a hypothesis that these therapies could potentially have an effect. In the future, large-scale clinical cohort studies are needed to assess the clinical value of intravenous ribavirinin pediatric adenovirus infection. There is limited research on therapeutic drug monitoring (TDM) of ribavirin, and further research can be conducted to explore ribavirin TDM in depth. This research can focus on how to standardize intravenous administration of ribavirin to balance effectiveness and safety considerations.

## Conclusion

This is the first report of ribavirin intravenous combined with IFNα1b aerosol inhalation in the treatment of severe adenovirus pneumonia in children. It provides a new treatment option for similar cases. Enhanced research and clinical endeavors are imperative to optimize the therapeutic regimen of ribavirin for HAdV infection, aiming to furnish comprehensive pharmacological insights for the management of adenovirus infection in pediatric patients.

## Data Availability

The original contributions presented in the study are included in the article/Supplementary Material, further inquiries can be directed to the corresponding author.
